# Field reconnaissance data from GEER investigation of the 2018 M_W_ 7.5 Palu-Donggala earthquake

**DOI:** 10.1016/j.dib.2021.106742

**Published:** 2021-01-15

**Authors:** Jack Montgomery, Joesph Wartman, A. Nicole Reed, Aaron P. Gallant, Daniel Hutabarat, H. Benjamin Mason

**Affiliations:** a Auburn University, Auburn, AL, USA; b University of Washington, Seattle, WA, USA; c University of Maine, Orono, ME, USA; d University of California, Berkeley, CA, USA; e Oregon State University, Corvallis, OR, USA

**Keywords:** Flowslide, Landslide, Liquefaction, Ground failure, Digital surface model, Unmanned aerial vehicle (UAV)

## Abstract

The M_w_7.5 Palu-Donggala earthquake occurred on 28 September 2018 and caused significant damage in Palu City and the surrounding Central Sulawesi region of Indonesia. The earthquake initiated a series of catastrophic landslides (classified as *flowslides*) [1,2], collapsed buildings, and generated tsunami waves that impacted Palu Bay's coast. The earthquake claimed over 4000 lives, making it the deadliest natural disaster of 2018. We performed a post-earthquake field reconnaissance and collected perishable data at the sites of five significant flowslides (named for the communities where they occurred: *Balaroa, Petobo, Lolu Village, Jono Oge*, and *Sibalaya*), as well as at other damage locations in the mesoseismal region. Our field team consisted of five U.S.-based members, who were sponsored by the U.S. National Science Foundation-supported Geotechnical Extreme Events Reconnaissance (GEER) organization [3], in collaboration with geologists, geotechnical engineers, and other researchers from Indonesia's Center for Earthquake Studies (PusGen) and the Indonesian Society of Geotechnical Engineers (HATTI) [this international team is collectively referred to as the Palu Earthquake “;*GEER*” team]. The GEER team arrived at Palu City on 13 November 2018 and conducted five days of extensive fieldwork using instrumentation from the Natural Hazards Reconnaissance Facility (known as the “RAPID”) [4,5], including mobile data collection software, digital imaging systems, high-resolution Global Navigation Satellite System (GNSS) antennas, and unmanned aerial vehicles (UAVs, or “;drones”). The resulting dataset includes over 2000 geotagged photographs, UAV images, ground coordinates, and other field measurements and observations, as well as associated post-processed geospatial data products (point clouds, digital surface models, orthomosaic images). Additionally, we used remote sensing data (i.e., pre- and post-event satellite imagery) to generate displacement vectors for over 1200 structures affected by the flowslides. The complete reconnaissance dataset is openly available on DesignSafe [6]. The data collected by the field team and subsequent mapping efforts, which document the morphology and patterns of movements of the flowslides, may be used by researchers studying liquefaction-induced flowslides. In addition, the displacement mapping provides a unique dataset for researchers who are calibrating and verifying simulation models of landslide displacements, or who are seeking a validation dataset for image correlation analysis (including machine learning routines). This dataset is associated with original research presented in “;East Palu Valley Flowslides Induced by the 2018 M_W_ 7.5 Palu-Donggala Earthquake” [1] and also is the basis of research presented by Gallant et al. [2].

## Specifications Table

**Table:** 

Subject	Geotechnical Engineering and Engineering Geology
Specific subject area	Earthquake reconnaissance, flowslide, landslide, liquefaction, ground failure, digital surface model, unmanned aerial vehicle (UAV), remote sensing, photogrammetry, geotechnical earthquake engineering
Type of data	High-resolution photographs Orthomosaic images Point clouds Digital surface models Vector data
How data were acquired	Field reconnaissance was performed with a range of instrumentation including GPS receivers (base-and-rover configuration of Leica GS18 survey-grade GNSS RTK system; and a Garmin GPSMAP 64 handheld GPS unit), and unmanned aerial vehicles (DJI Phantom 4 Pro V2, DJI Mavic 2 Pro, and DJI Inspire 2 platform with a Zenmuse X4S camera). Displacement mapping was performed using satellite imagery provided by DigitalGlobe's Open Data Program [7].
Data format	Photographs (JPEG, RAW), GPS tracks (GPX), displacement maps (Shapefile), orthomosaic maps (GeoTiff), point clouds (LAS), and digital surface models (GeoTiff).
Parameters for data collection	Data were collected at five flowslide sites and other locations in and around Palu City. The primary focus was documenting the surface morphology of the flowslide areas, as well as damage to buildings and other infrastructure in the region.
Description of data collection	Team members took photographs to document damage from the earthquake. UAV flights were conducted at five flowslides to obtain aerial imagery, which was post-processed using the photogrammetry software Pix4D to generate point clouds and orthomosaic maps. The displacement of individual structures was measured by manually locating features in imagery collected before and after the earthquake.
Data source location	Palu City and surrounding areas, Central Sulawesi, Indonesia All photos are geotagged with location information. See Figure 1 for a map showing survey locations.
Data accessibility	Repository name: DesignSafe Data Depot Data identification number: https://doi.org/10.17603/ds2-q22d-bk96 Direct URL to data: https://www.designsafe-ci.org/data/browser/public/designsafe.storage.published//PRJ-2903
Related research article	H.B. Mason, J. Montgomery, A.P. Gallant, D. Hutabarat, A.N. Reed, J. Wartman, M. Irsyam, P.T. Simatupang, I.M. Alatas, W.A. Prakoso, D. Djarwadi, R. Hanifa, P. Rahardjo, L. Faizal, D.S. Harnanto, A. Kawanda, A. Himawan, and W. Yasin, East Palu Valley Flowslides Induced by the 28 September 2018 MW 7.5 Palu- Donggala Earthquake, Geomorphology. 373 (2021), 107482. https://doi.org/10.1016/j.geomorph.2020.107482

## Value of the Data

• The openly available reconnaissance data collected by the GEER team preserve a unique, highly perishable dataset on flowslides initiated by the deadly 2018 Palu-Donggala earthquake. Documenting and recording the earthquake's impacts is critical for developing lessons and formulating policies that may prevent loss of life in future seismic events.
• These data are useful to the broader geotechnical engineering and engineering geology communities, especially those studying liquefaction-induced flowslides and their consequences.
• The data document the patterns, styles, and scale of movements and the associated surface morphologies of the flowslides. Researchers may use this information to verify and calibrate models that simulate the flowslide initiation process and consequent displacement behavior. The data may also serve as a validation dataset for image analysis, including machine learning algorithm training. Finally, the data may be used to guide future subsurface investigation efforts in the Palu region to better understand the soil conditions that lead to these flowslides.
• The geospatial and mapping data may be used to support rebuilding and recovery in the region, and develop seismic hazard microzonation and land use policy maps.

## 1 Data Description

**Fig. 1:**
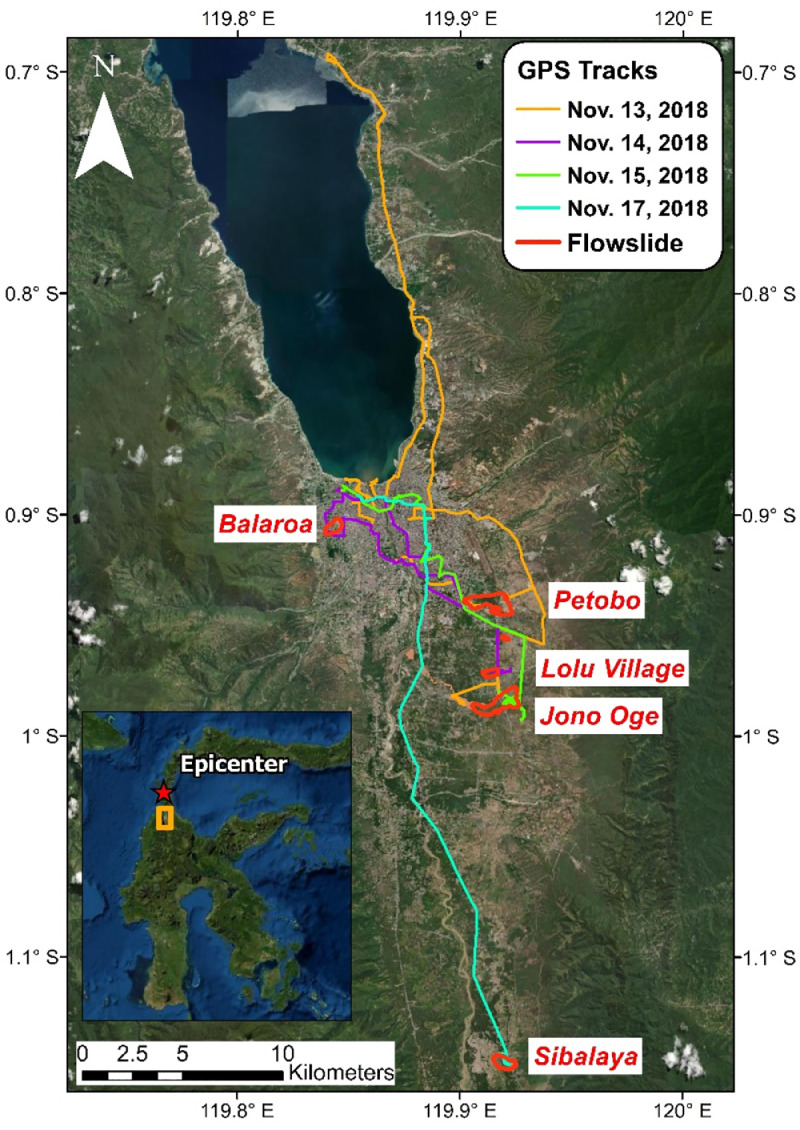
Areas surveyed by the GEER Team along with flowslide locations. GPS tracks were not available for November 16. Satellite imagery from ESRI [9].

**Fig. 2:**
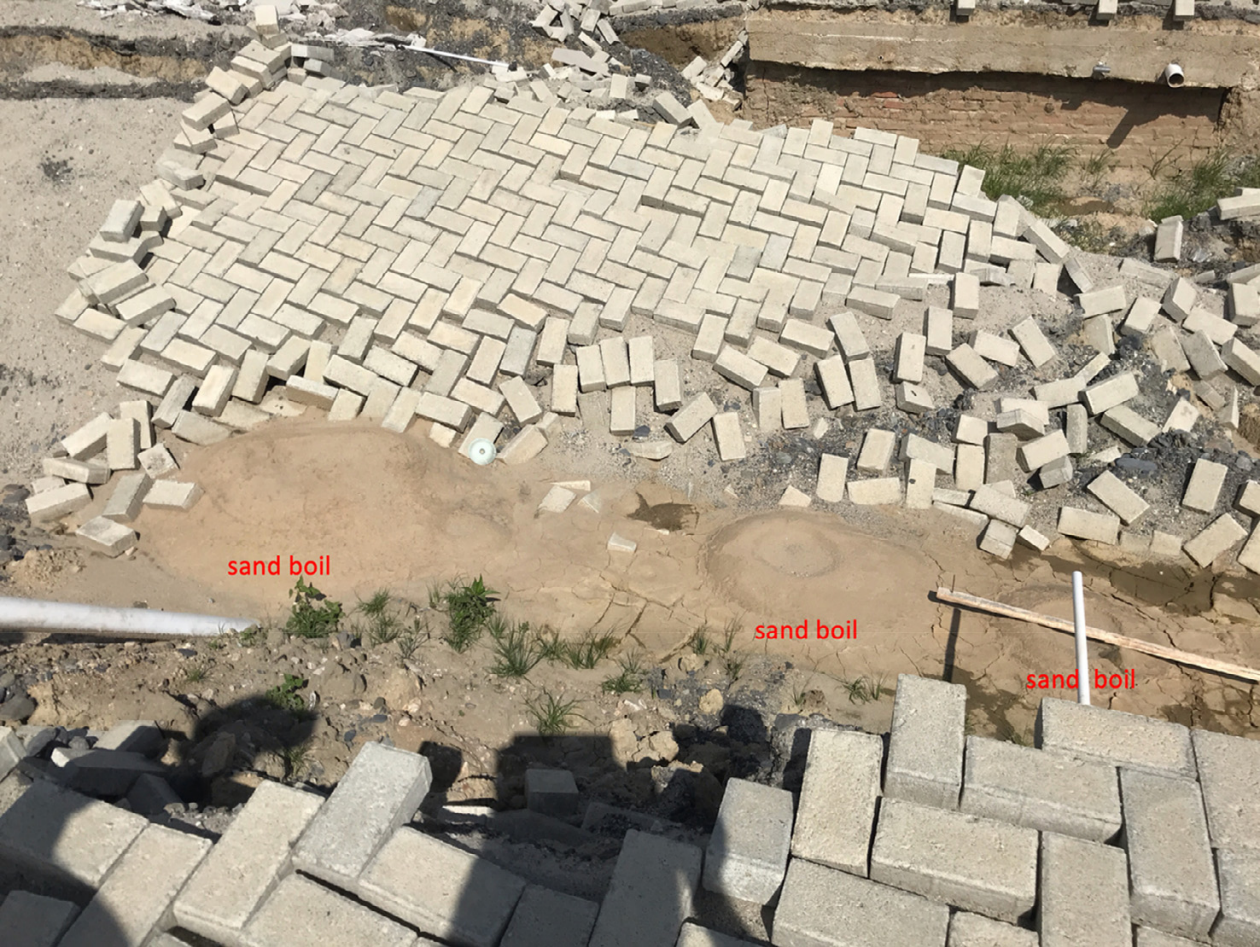
Example of three cone-shaped sand boils indicating soil liquefaction.

**Fig. 3:**
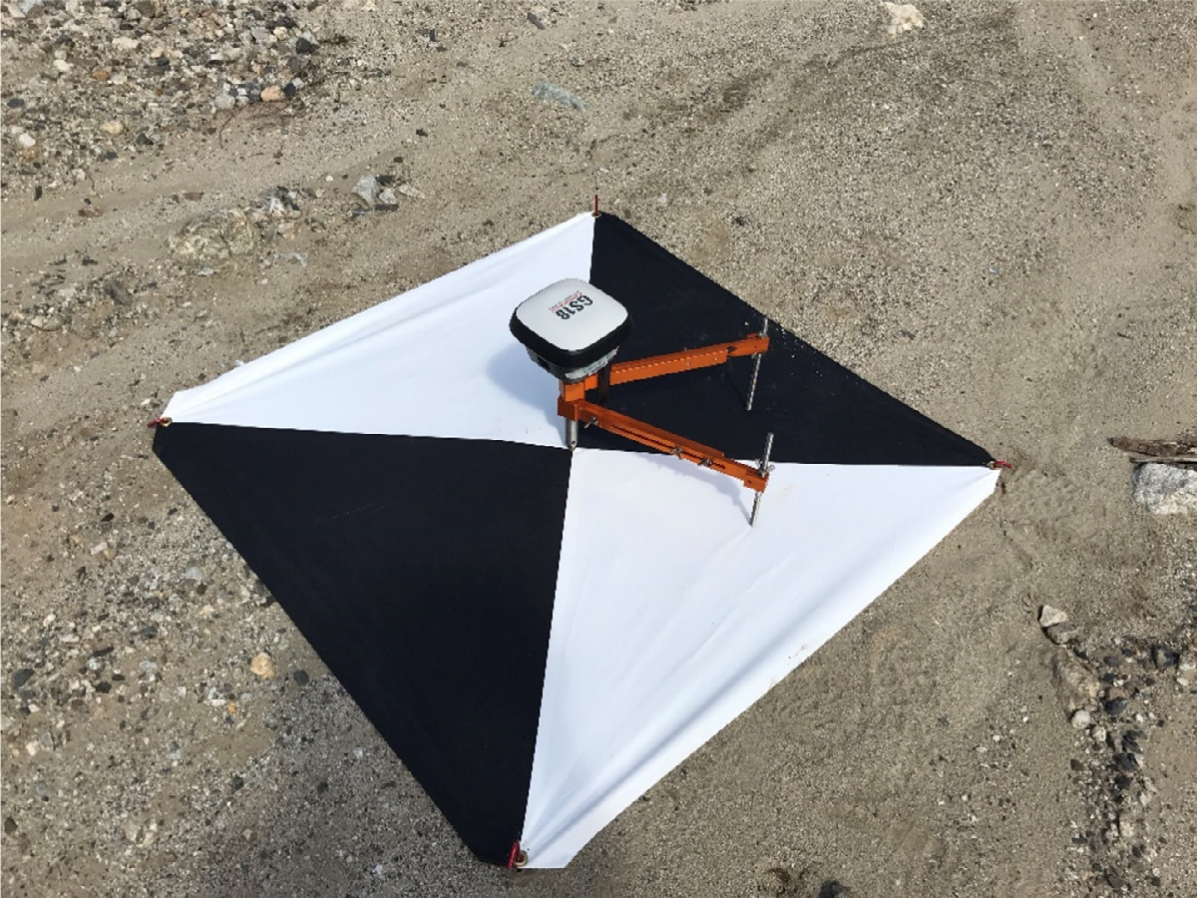
Photograph of a typical ∼1 m^2^ ground control points (GCP) target. The vinyl targets were secured to the ground using tent stakes. The photograph also shows the Leica GS-18 GNSS antenna on an orange colored portable “;spike mount” used to measure the precise center location of the GCP.

**Fig. 4:**
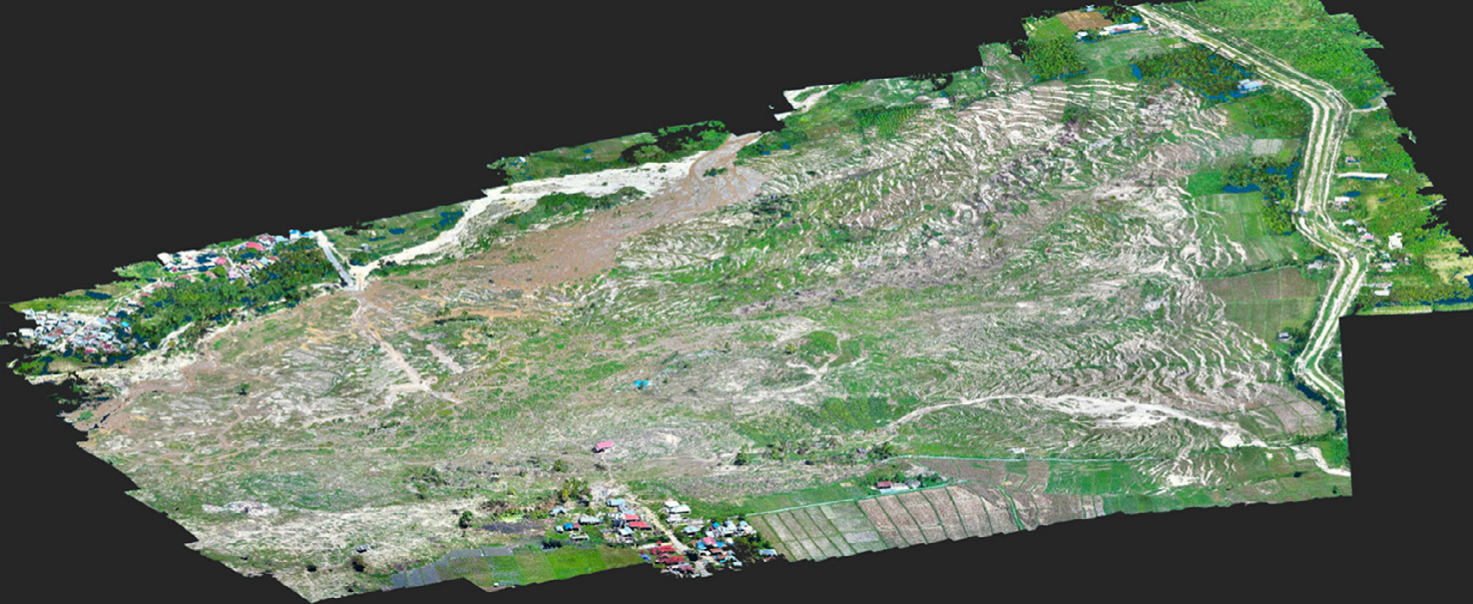
UAV image-derived point cloud for a portion of the Jono Oge flowslide. The Pix4Dmapper SfM algorithm assigns a realistic color to each point based on color returns in the raw UAV images. The point cloud is displayed in the open-source software CloudCompare.

**Fig. 5:**
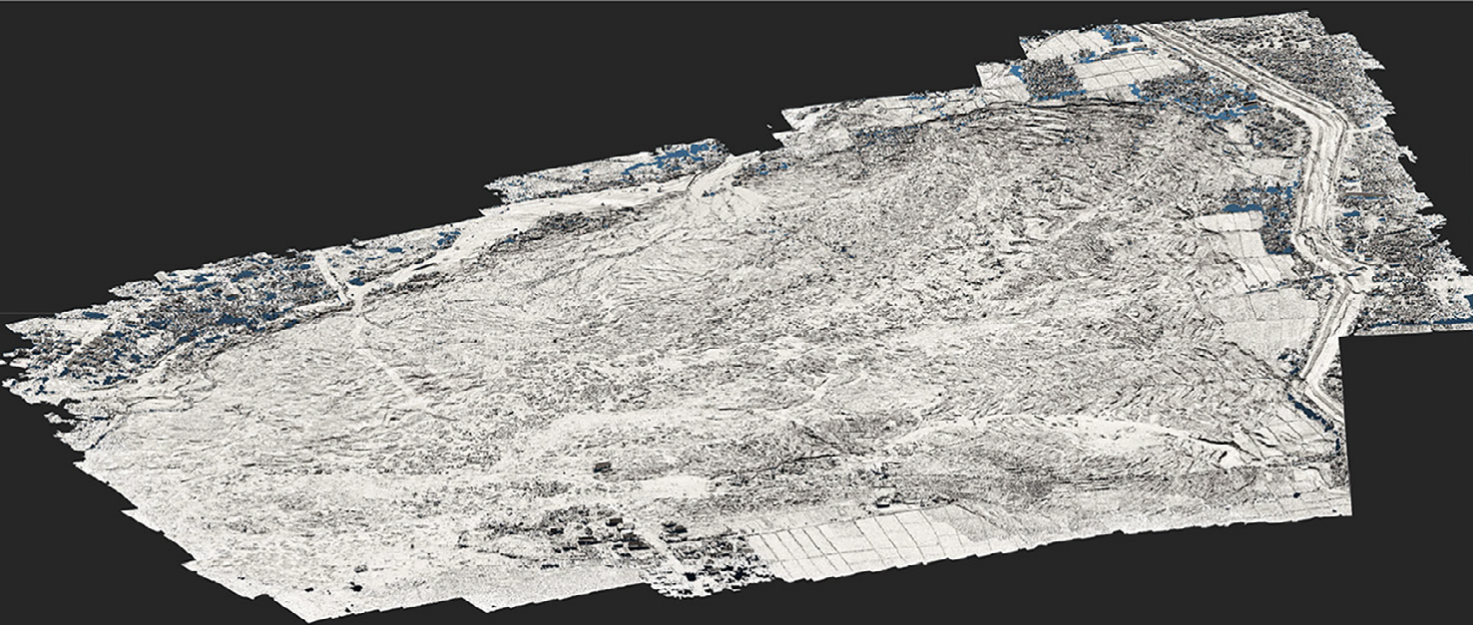
UAV image-derived digital surface model (DSM) the portion of the Jono Oge flowslide shown in Fig. 4. The shaded relief mapping, which accentuates morphological features, was created in the software CloudCompare. Areas colored blue indicate regions where vegetation concealed the ground surface.

**Fig. 6:**
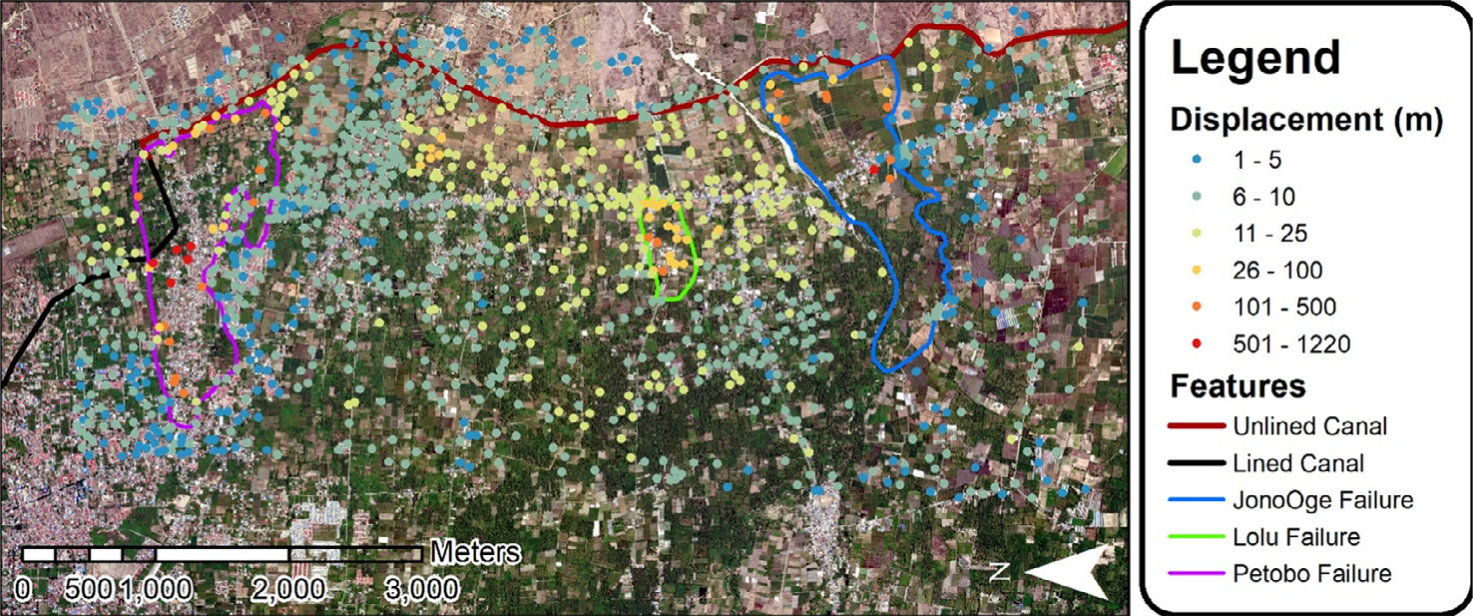
Distribution and magnitude of structure displacements shown on pre-earthquake satellite images.

**Fig. 7:**
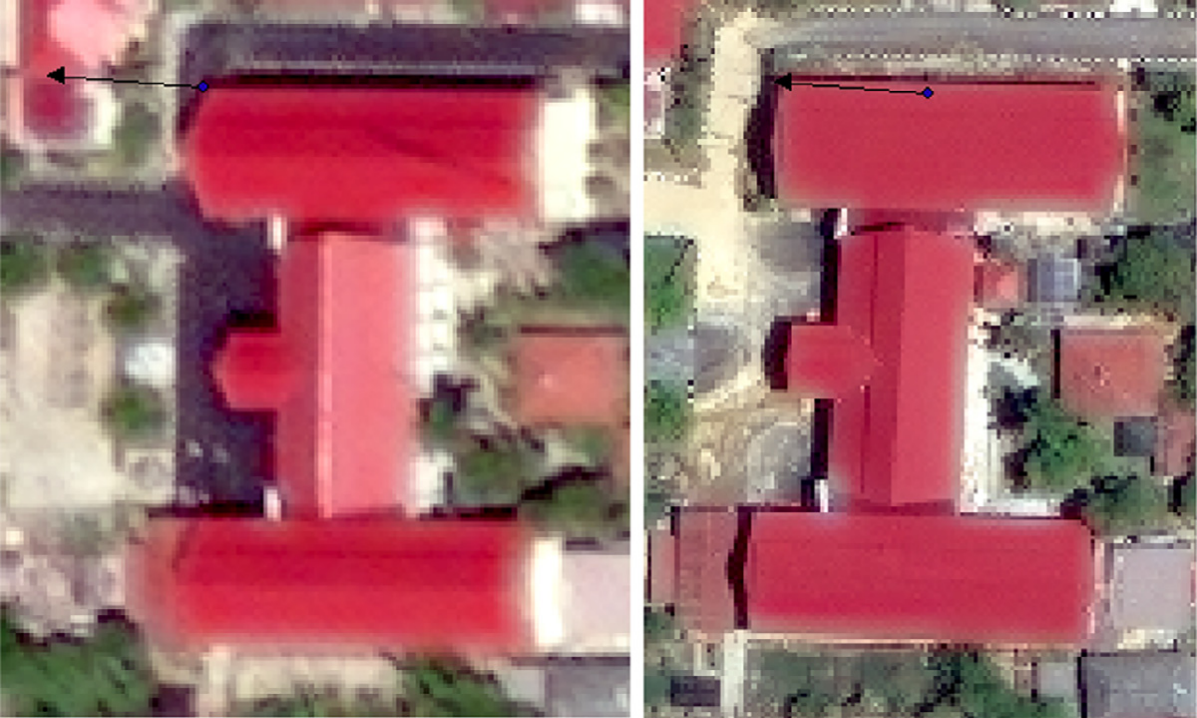
Ideal building for mapping from (left) before and (right) after the earthquake in ArcMap.

**Fig. 8:**
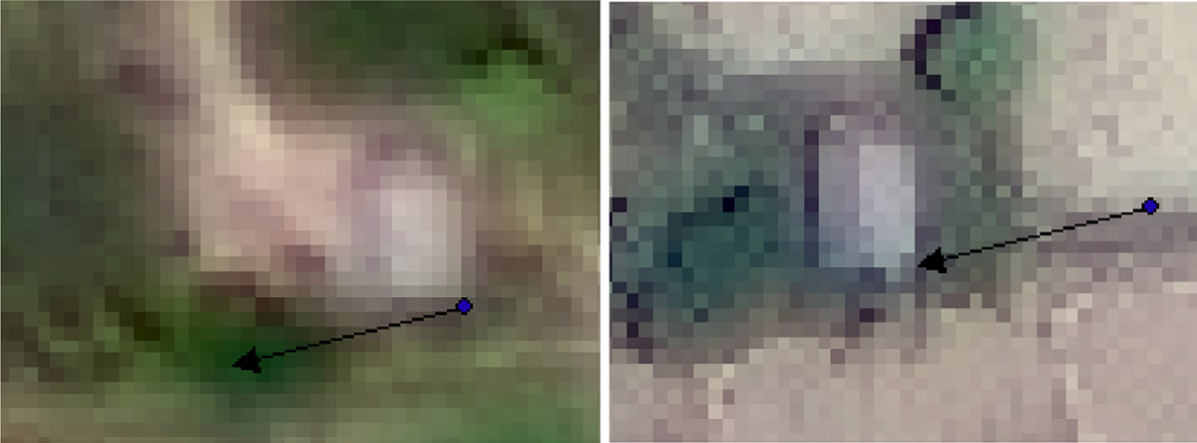
Suboptimal building for mapping from (left) before and (right) after the earthquake in ArcMap.

**Fig. 9:**
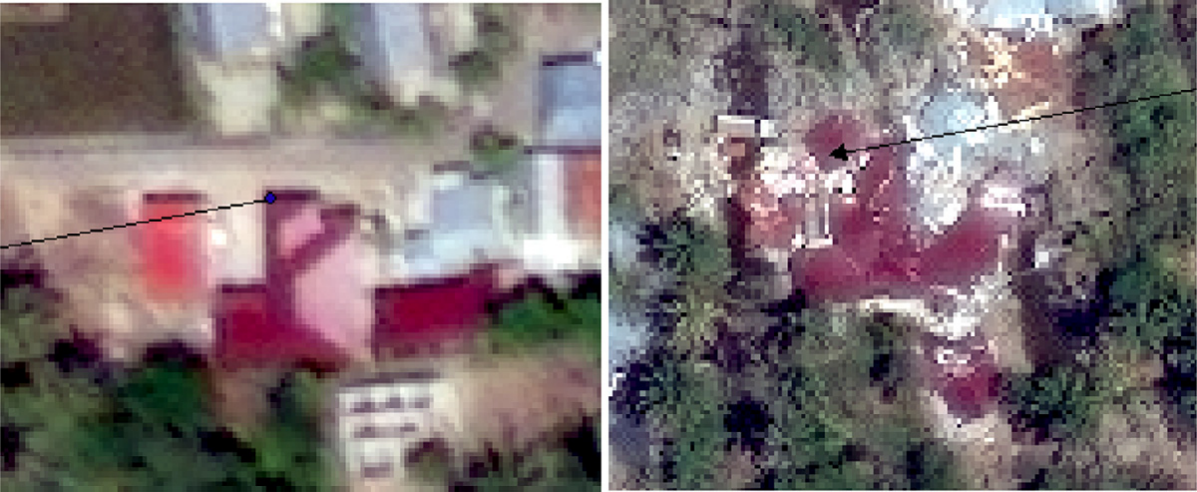
Unique roof from (left) before and (right) after the earthquake in ArcMap.

**Fig. 10:**
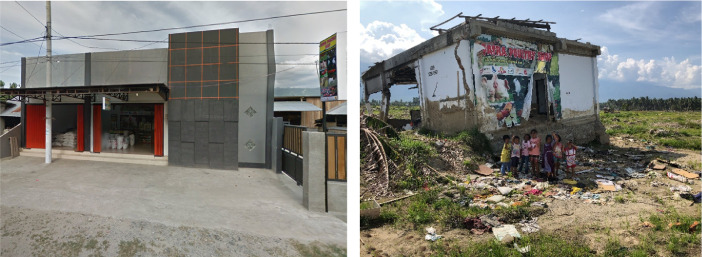
Patra Poultry Shop in Jono Oge from (left) before the earthquake in Google Streetview and (right) after the earthquake as observed by the reconnaissance team. The children's faces in the photograph taken by the reconnaissance team are blurred to protect their privacy.

The GEER field investigation took place in Central Sulawesi from 13 to 17 November 2018 (Fig. 1). The reconnaissance was followed by post-processing of the field data, including the development of digital surface models and orthomosaics derived from the UAV images. In addition, other openly available data such as satellite imagery were used to map displacements of structures impacted by the flowslides. The data collected by the GEER team is available on DesignSafe [6], and the findings of related research are presented by Mason et al. [1, 8] and Gallant et al. [2]. The archived data is organized into three general categories. The first category includes geotagged photographs collected by individual team members using handheld cameras, the mobile software application RApp [4], and UAVs. These photographs are organized into folders by the last name of the team member that took the photographs and then by location (e.g., Jono Oge) or category (e.g., sand boils as shown in Fig. 2). The specific location coordinates for each photograph are stored in the metadata (exchangeable image file format, or EXIF) of each image file. The second category of data includes post-processed, UAV-derived products, such as orthomosaic images, point clouds, and digital surface models. Ground control points (GCPs, Fig. 3) were used for surveys at Lolu Village, Jono Oge, and Sibalaya. Three-dimensional point clouds created from the UAV images (e.g., Fig. 4) are archived as standard LAS (”LASer format”) files. The orthorectified orthomosaic images and digital surface models (e.g., Fig. 5) are stored as GeoTIFF files for the major flowslides. The final category is geospatial data in geographic information systems (GIS) standard shapefile format. These geospatial data include GPS tracks collected by the team (shown in Fig. 1) and the satellite imagery-derived displacement maps (Fig. 6). GPS tracks were not collected on November 16, but the team followed a similar route to November 15. Examples of structures used for the displacement mapping are shown in Fig. 7, Fig. 8, Fig. 9, Fig. 10.

## 2 Experimental Design, Materials and Methods

Before deploying to the field, the team identified sites of interest by reviewing news and press reports, social media postings, and satellite images. Upon arrival, the GEER team first conducted a brief survey of Palu Bay's coastal areas impacted by the tsunamis. The GEER team then focused on detailed documentation of four of the five large flowslides that occurred in or near Palu City that had not been previously studied in the field (most of the field data and evidence for the fifth flowslide, Balaroa, was altered or removed before we arrived in Palu City). The GEER team's primary objectives were to collect and document perishable data that may be used to understand the sequence of events and general mechanisms resulting in these large landslides. Details of the data collection process are provided in the following sections.

### 2.1 Field reconnaissance and photographs

Each GEER team member travelled with smartphones and/or tablets (with a beta version of the mobile data collection software RApp [4]), which were used to take photographs of key earthquake and flowslide damage features in the region. Our effort focused on recording damage to structures, roadways, an irrigation canal that ran along the eastern edge of the valley, and to agricultural fields found throughout the flowslide region. Special attention was given to documenting field evidence of sand boils (Fig. 2), which form in response to high pore water pressures within the ground and thus serve as direct evidence of liquefaction. While much of the reconnaissance effort focused on the flowslides, we also observed and photographed other secondary features of the earthquake, including tsunami damage and non-liquefaction-related coseismic landslides.

### 2.2 Collection and processing of UAV imagery

We used several commercial-grade UAVs during the reconnaissance; however, nearly all of the high-resolution mapping was conducted using a DJI Inspire 2 platform with a Zenmuse X4S camera (1-in. sensor, with 20-megapixel resolution). The UAV flights were typically flown at an elevation of 65 m (with nadir images having 75% overlap), providing a ground sampling distance pixel resolution of ∼2 cm. Flight missions were flown in the autonomous mode using the mobile (iOS) application Pix4Dcapture. The accuracy of UAV surveys was enhanced using GCPs at Lolu Village, Jono Oge, and Sibalaya. High resolution real-time kinematic GNSS surveys (Leica GS-18) were used to determine the GCP's precise coordinates, which were marked on the ground with high contrast aerial targets (Fig. 3). Typically, 8 to 12 well-distributed GCPs were used at each for flowslide site survey. The GNSS logs (rate: 1 sample/s for > 2 h) were later post-processed in Leica Infinity software to improve the measurements' accuracy. The following steps were followed:

1. Obtain earth-centered, earth-fixed (9ECEF) coordinates for the base station through static GNSS baseline processing using a nearby continually operating reference station as a reference in Leica Infinity software. The precise ephemeris and NGS calibrated antenna models were used in this baseline processing.
2. Perform a local GNSS baseline processing at all GNSS rover positions (i.e., GCP locations) located within a few km from the base using Leica infinity software, referenced to the Base coordinates obtained in step 1. The precise ephemeris, L1/L2/L5 frequencies, and NGS calibrated antenna models were used.
3. Coordinates were projected from ECEF to UTM Zone 50 S (WGS84) referenced to the EGM08 geoid model.

The UAV images were then processed with the precise GCP locations in the structure-from-motion (SfM) photogrammetric software Pix4Dmapper to generate georectified orthomosaic images, point clouds, and digital surface models (DSM). The point clouds in the dataset are not filtered for anthropogenic objects (buildings, infrastructure) or vegetation, and therefore represent ground surfaces (i.e., DSM) rather than ground elevations. Nevertheless, the flowslides stripped much of the vegetation from the sites and buried many buildings, so much of the immediate post-event landscape was, in effect, a “;bare earth” landscape. Fig. 4 shows a portion of an example point cloud for a flowslide, while Fig. 5 shows a shaded relief digital surface model.

### 2.3 Displacement mapping using satellite imagery

The displacement mapping for this project was performed using satellite images provided by DigitalGlobe's Open Data Program. DigitalGlobe provides open access to pre- and post-event images for natural disasters to support response and recovery efforts. For this study, the pre-event image (I.D. 1030010078CD4A00), was taken on 20 February 2018, while the post-event image (I.D. 1040010042376D00) was taken four days after the earthquake on 3 October 2018. The satellite images were used in the GIS software program ArcMap [9]. A shapefile was created using a projected coordinate system (WGS 1984 UTM zone 50S) so that measurements could be taken. This shapefile was then populated by identifying a building's location in the pre- and post-event images and measuring the amount of displacement that had occurred. This mapping process was completed for 1220 structures (Fig. 6).

To measure the movement of a building, a vertex or another well-defined point was chosen on both images. The precision of the point chosen was especially important for buildings with small (<25 m) displacements since measurement error could skew the data. Fig. 7 shows a clearly identifiable structure. The pre-earthquake location is marked with a colored dot, and the arrow terminates at the post-earthquake displaced location. The uncertainty of this displacement measurement is low because the building shape is unique, its movement was minimal, and perhaps most importantly, the building was recorded by a high-resolution satellite image both before and after the event. However, it was not always possible to obtain such high-quality displacement measurements, particularly for sparsely populated or rural areas. There are relatively few structures to observe at these locations, and often the satellite images were of lower resolution. Fig. 8 shows an example of imagery where the footprint of a rural structure was not as well defined, but of adequate quality to interpret displacements. For such cases where the building vertices were vague, a corner was assumed by following the more defined sides of the roof to where their intersection would have been depicted if the imagery had better resolution.

There were additional difficulties with the areas that experienced large (>25 m) displacements. Identifying buildings is more challenging, as they did not merely slide in a uniform, coherent manner but were often rotated in the flowslide along a nonlinear path. To identify buildings in these areas, we focused on structures with unique roof lines and colors (Fig. 9). Buildings that could not be confidently identified from the before and after images were excluded. Within the footprint of the flowslides, the buildings were often too heavily damaged to be clearly identified in the post-event imagery. In these cases, the reconnaissance photographs and UAV imagery were used to supplement the satellite images. For example, the reconnaissance team photographed the damaged remains of the Patra Poultry Shop at the Jono Oge flowslide, which provided the post-earthquake location (Fig. 10). Google Maps and Streetview was then used to locate the market's pre-earthquake position (Fig. 10), which was 1.2 km away from the final location. This finding allowed other structures, including a heavily damaged church, to be identified in the post-earthquake imagery.

## Ethics Statement

Not applicable.

## CRediT Author Statement

All: Investigation, Writing – Review & Editing; **Jack Montgomery:** Conceptualization, Methodology, Writing – Original Draft, Visualization, Data Curation; **Joseph Wartman:** Conceptualization, Methodology, Writing – Original Draft, Visualization, Data Curation; **A. Nicole Reed:** Methodology, Visualization; **Aaron P. Gallant:** Conceptualization, Methodology; **Daniel Hutabarat:** Conceptualization, Methodology; **H. Benjamin Mason:** Conceptualization, Methodology, Project Administration.

## Declaration of Competing Interest

The authors declare that they have no known competing financial interests or personal relationships which have, or could be perceived to have, influenced the work reported in this article.
